# Evaluation of preoperative apical periodontitis, treatment indications, and methods in endodontically treated teeth: a retrospective study

**DOI:** 10.1186/s12903-025-05916-4

**Published:** 2025-05-09

**Authors:** Hüseyin Gündüz, Züleyha Baş, Aslı Zeynep Kapoğlu Kılıç, Beyda Sevgül Aparı, Pelinsu Şahin

**Affiliations:** 1 Department of Endodontics, Faculty of Dentistry, Bilecik Şeyh Edebali University, Bilecik, Turkey; 2 Department of Endodontics, Faculty of Dentistry, Van Yuzuncu Yıl University, Van, Turkey

**Keywords:** Apical periodontitis, Endodontic treatment, Epidemiology, Oral health

## Abstract

**Background:**

This study evaluated the presence of preoperative apical periodontitis (AP) in endodontically treated teeth within a Turkish population, along with its causes, treatment methods, and the effectiveness of preventive and early intervention practices.

**Methods:**

A retrospective analysis was conducted on 1,440 teeth from 1,055 patients treated at Van Yüzüncü Yıl University between 2021 and 2023. Preoperative panoramic and periapical radiographs and postoperative periapical radiographs were examined. Data recorded included patient demographics, treated tooth location, presence of preoperative AP, coronal restorations, reasons for treatment, treatment methods, and number of missing and endodontically treated teeth. Statistical analyses were performed using Chi-Square, Mann-Whitney U, Kruskal-Wallis, and Spearman correlation tests.

**Results:**

The overall incidence of AP was reported as 28.7%. It was more frequently observed in the mandible than in the maxilla and in incisors compared to other tooth groups (*p* < 0.001). Caries was the primary reason for treatment in molars (81.5%), while periodontal disease was more common in incisors (*p* < 0.001). As age increased, the number of endodontically treated and missing teeth also rose (*p* = 0.019; *p* < 0.001). Teeth with crowns had a lower AP rate, while retreatments due to periodontal disease or previous root canal failures showed higher AP rates (*p* < 0.001).

**Conclusions:**

The high AP rate and the predominance of caries and periodontal disease as treatment causes indicate insufficient application of preventive and early treatments. AP prevalence varied by jaw location, tooth group, coronal restoration and treatment method. This study provides epidemiological data on endodontically treated teeth and their association with AP. These findings emphasize the importance of early diagnosis, preventive measures, and effective treatment planning in preserving tooth survival.

## Introduction

Pulp infection is a common oral health problem caused by various factors, including dental caries, periodontal diseases, prosthetic treatments, trauma, and inadequate restorative practices. The incidence of dental caries is influenced by multiple factors such as age, gender, oral hygiene habits, and socioeconomic status [1]. Dental caries is recognized as one of the primary causes of endodontic treatment, as it induces irritation in the dental pulp and periradicular tissues. Consequently, endodontic treatment should be planned based on an accurate diagnosis of the existing condition [2].

Apical periodontitis (AP) results from primary root canal infections following pulp necrosis or reinfections due to coronal leakage or persistent infection after root canal treatment [3]. The primary goal of endodontic treatment is to eliminate or minimize the microbial load within the root canal system through chemo-mechanical debridement and effective canal filling [4]. However, despite technological advancements in root canal therapy, an increase in the prevalence of AP has been reported [5].

Endo-perio lesions arise due to the close connection between the pulp and periodontal tissues, allowing infections to spread between them [6]. These lesions can mimic either endodontic or periodontal disease, making diagnosis challenging [7]. Identifying the primary source of infection is crucial, as treatment success depends on a targeted approach. A clear understanding of this intercommunication helps clinicians choose the most appropriate treatment strategy, leading to better patient outcomes [8].

Radiographic evaluations play a critical role in ensuring accurate diagnosis in endodontic treatment planning, alongside clinical assessments. Panoramic and periapical radiographs are essential tools for assessing the condition of the pulp and periapical tissues, supporting both diagnostic and therapeutic processes [9, 10]. Panoramic radiography, frequently used in routine dental practice, is a low-dose, cost-effective method for a comprehensive oral health assessment [11]. It provides an overview of the maxillary and mandibular jaws and their supporting structures in a single image, offering valuable information on periapical tissues, restoration types, missing teeth, and the number of teeth treated with root canal therapy [12]. Periapical radiographs, on the other hand, offer greater sensitivity than panoramic radiographs for detailed assessments of treatment outcomes and periapical lesions [13]. However, both periapical and panoramic radiographs are limited by their two-dimensional nature, which may lead to inaccuracies in evaluating lesion size and complex root canal anatomy [14]. Cone-beam computed tomography (CBCT), with its three-dimensional imaging capability, overcomes these limitations, providing enhanced diagnostic accuracy. Nevertheless, due to its higher radiation dose, CBCT should be reserved for cases where conventional two-dimensional imaging proves insufficient [15].

Epidemiological studies that document detailed endodontic treatment records are crucial for evaluating and improving knowledge about the incidence and distribution of patients requiring endodontic care within specific populations [16, 17]. Demographic data play a key role in epidemiological studies, providing insights into endodontic treatment patterns and serving as a guide for healthcare planning [18, 19]. Understanding the factors influencing endodontic treatment is essential for effectively guiding health policies and optimizing resource allocation [20]. This study aims to contribute significantly to the development of health policies and the efficient management of health services by evaluating endodontic treatment needs and distribution within a Turkish subpopulation. Given that epidemiological data may change over time due to various influences, including advancements in healthcare practices and shifting population dynamics, it is crucial to ensure that such information remains up to date [21].

The literature indicates that the primary goal of endodontic treatment is to prevent or eliminate AP and that preoperative AP significantly influences treatment outcomes [4, 22, 23]. Therefore, understanding the factors influencing AP, which affect the objectives and success of endodontic treatment, is crucial for treatment prognosis and planning [24]. Additionally, analyzing the relationship between the number of endodontically treated teeth and missing teeth provides valuable insights into the survival of endodontically treated teeth [25].

Previous studies worldwide have investigated the prevalence of AP and its impact on endodontic treatment outcome [5, 21]. However, these studies have primarily relied on retrospective analyses of teeth that underwent endodontic treatment. One of the major limitations of retrospective studies is the uncertainty regarding the pre-existing status of AP, its healing process, and the timing of treatment. In contrast, in this study, the pathological nature of AP was confirmed through clinical and radiographic evaluations, ensuring a more accurate assessment of its presence and its relationship with endodontic treatment [5, 26].

In Turkey, research on the epidemiology of endodontic treatment is limited [17, 27–32]. Existing studies were conducted on specific patient groups with relatively small sample sizes and focused solely on AP as a consequence of endodontic treatment. Additionally, most studies have primarily focused on the radiographic assessment of endodontically treated teeth without providing clear information regarding the timing of treatment or the preoperative condition of the teeth [28, 30, 31, 33]. To address these limitations, this study aims to provide updated epidemiological data on the Turkish population by comprehensively analyzing the preoperative conditions of patients undergoing endodontic treatment through clinical and radiographic evaluations. Specifically, it seeks to assess the prevalence of AP, its association with endodontic treatment, and the reasons for treatment, as well as the treatment methods applied to teeth requiring root canal therapy in a Turkish subpopulation.

### Hypotheses

- The first null hypothesis suggests no difference in the preoperative presence of AP, reasons for treatment, and treatment methods based on gender, tooth location, and tooth group.
- The second null hypothesis states that previous restorations, reasons for treatment, and treatment methods do not impact the presence of AP.
- The third null hypothesis assumes no relationship between the number of endodontically treated teeth and the number of missing teeth.

## Materials and methods

This study was conducted in accordance with the guidelines for reporting observational studies, as outlined in the STROBE (Strengthening the Reporting of Observational Studies in Epidemiology) statement [34]. This retrospective study was performed in accordance with the Declaration of Helsinki. The study was approved by the Non-Interventional Ethics Committee of Van Yüzüncü Yıl University (approval no: 2024/05–07). This retrospective study was conducted using anonymized patient data, and therefore, the requirement for obtaining individual informed consent was waived by the Non-Interventional Ethics Committee of Van Yüzüncü Yıl University. The study evaluated teeth treated at the Department of Endodontics, Faculty of Dentistry, Van Yüzüncü Yıl University, between June 2021 and June 2023.

Preoperative panoramic and periapical radiographs, as well as postoperative periapical radiographs, were assessed. Panoramic radiographs were acquired using a digital Orthophos XG3 device (Dentsply Sirona, Bensheim, Germany) with a beam area of 0.325 × 13 cm², operating at 67 kVp, 13 mA, and an exposure time of 14.1 s. Periapical radiographs were obtained using a digital Planmeca Prosensor (Planmeca, Roselle, IL, USA) with a 4.5 × 5.5 cm sensor, operating at 66 kV and 0.8 mA, using the paralleling technique.

### Sample size calculation

The sample size was calculated using G*Power 3.1 (Heinrich, Heine University) software based on the study by Meirinhos et al. [35]. The parameters considered were as follows: Significance level α = 0.05, power = 0.85, effect size = 0.084. It was determined that the total number of samples should be 1273 in order to detect a significant difference between the groups. The total number of samples was determined as 1440, considering the 10% possible losses. A total of 24,693 teeth from 1,055 patients were examined, and it was determined that 1,440 teeth underwent root canal treatment.

### Inclusion and exclusion criteria

#### Inclusion criteria

- Patients aged between 15 and 80 with preoperative panoramic and periapical radiographs, postoperative periapical radiographs, and accessible demographic data.
- Patients who underwent endodontic treatment following clinical and radiographic examinations between June 2021 and June 2023.

#### Exclusion criteria

- Patients with blurred radiographic images in the anterior region due to exposure errors or patient movement.
- Radiographic images with distortion, magnification, or artifacts.
- Impacted teeth and third molars.
- Teeth that have undergone surgical treatments such as root resection or hemisection.

**Fig. 1:**
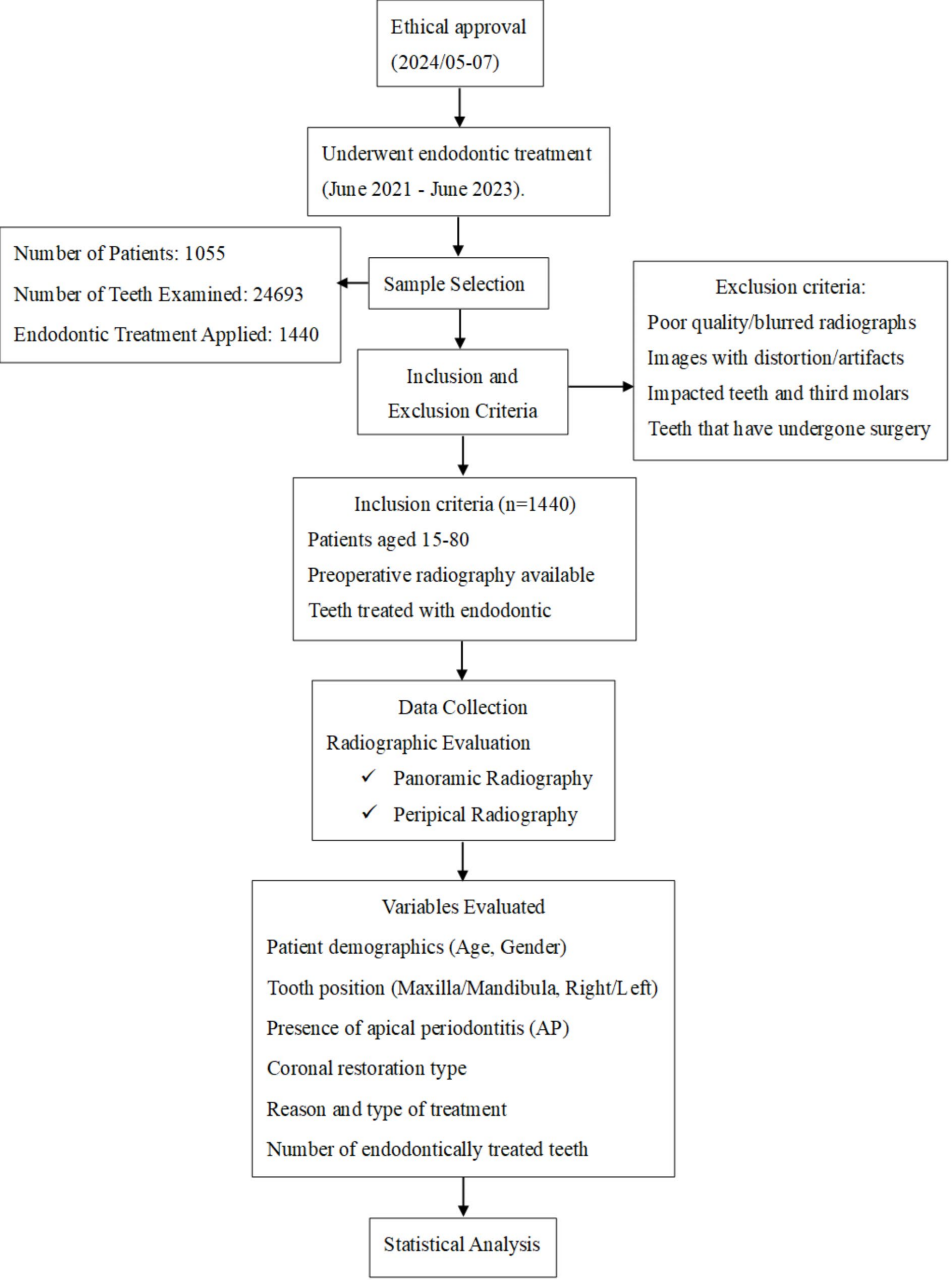
Flow chart illustrating the study flow of teeth meeting the inclusion criteria

A flow chart illustrating the inclusion criteria for teeth evaluated in this study is shown in Fig. 1.

### Data collection

Radiographs were reviewed by two endodontists with three years of experience. The observers underwent a calibration process, which involved reviewing 100 standard radiographs previously scored by the index developers. Any discrepancies in their assessments were resolved through discussion.

The following variables were recorded:

- Patient demographics: Age and gender.
- Tooth location: Classified as mandibular or maxillary, and right or left quadrant.
- Presence of AP: Defined as a periapical radiolucency exceeding the normal width of the periodontal ligament space.
- Type of coronal restoration: Categorized as none, amalgam, composite, crown, or bridge.
- Reasons for treatment: Including caries, failed root canal treatment, periodontal disease, prosthetic reasons, or trauma. Endodontic treatment is performed before prosthetic procedures in cases where tooth position or alignment leads to pulp exposure or a high risk of perforation during preparation. This approach also helps in managing sensitivity and discomfort in prepared teeth, ensuring long-term prosthetic success.
- Type of treatment: Identified as primary root canal treatment or retreatment.
- Number of missing teeth and endodontically treated teeth: Recorded.

Tooth position, coronal restoration, and the number of missing and endodontically treated teeth were recorded using panoramic radiographs taken during routine dental treatment practices. Preoperative AP presence, reasons for treatment, and type of treatment were assessed from periapical radiographs, which provided more accurate results for AP evaluation than panoramic radiographs and allowed for the evaluation of preoperative root canal morphology. Postoperative periapical radiographs were acquired to evaluate the performed treatments, assess their adequacy, and facilitate long-term treatment follow-up. Teeth were categorized as endodontically treated if a radiopaque material was observed in the pulp chamber and/or one or more root canals.

### Periapical index

Periapical status was assessed radiographically using the Periapical Index (PAI) score [36]. Scores ranged from 0 to 5 as follows:

1. Normal periapical structures.
2. Minor changes in bone structure.
3. Changes in bone structure with slight mineral loss.
4. Periodontitis with well-defined bone circumscriptionand a halo of bone sclerosis.
5. Severe periodontitis with significant bone loss and a diffuse radiolucent image.

Scores of 1 and 2 indicated periapical health, whereas scores of 3, 4, and 5 represented AP. For multi-rooted teeth, the highest PAI score among the roots was considered. In cases of uncertainty, a consensus was reached, and the higher scores were selected. Importantly, during the assessment, observers were blinded to the patients’ identities and clinical status. AP cases were diagnosed based on the presence of at least one tooth with a periapical radiolucency measuring twice the width of the periodontal ligament space and a PAI score greater than 2 [37, 38].

### Statistical analysis

Data were analyzed using IBM SPSS Version 23 software. The normality of the data distribution was assessed using the Shapiro-Wilk and Kolmogorov-Smirnov tests. Categorical variables were analyzed using the Pearson Chi-Square test, with multiple comparisons conducted using the Bonferroni-adjusted Z-test. The Mann-Whitney U test was employed for pairwise comparisons of non-normally distributed variables. For comparisons involving three or more groups of non-normally distributed variables, the Kruskal-Wallis test was utilized, followed by the Dunn test for multiple comparisons. Relationships between non-normally distributed variables were examined using Spearman’s rho correlation coefficient and the significance level was *p* < 0.05.

## Results

### Reliability

The intraclass correlation coefficient values for intra-observer and inter-observer reliability exceeded 0.95, indicating excellent reliability.

### Descriptive statistics

A total of 1055 patients and 24,693 teeth were evaluated in this study. Root canal treatment was performed on 1440 teeth, with 4.2% of teeth having undergone previous root canal treatment. The average age of participants was 33.2 years, and 47.4% had at least one endodontically treated tooth. Newly performed endodontic treatment was most common in females (57.7%), in the maxilla (53.3%), on first molars (33.1%), on teeth without preoperative restorations (73.5%), and primarily due to caries (81.5%) (Tables 1 and 2).

**Table 1:** Frequency distributions and descriptive statistics of variables

	Frequency	Percentage (%)
Gender		
Male	457	43.3
Female	598	57.7
Jaw		
Mandible	673	46.7
Maxilla	767	53.3
Tooth position		
Mandible		
Right	351	52.2
Left	322	47.8
Maxilla		
Right	358	46.7
Left	409	53.3
Tooth groups		
Mandible		
First molar	281	41.8
First premolar	58	8.6
Second molar	143	21.2
Second premolar	109	16.2
Canine	31	4.6
Lateral incisor	28	4.2
Central incisor	23	3.4
Maxilla		
First molar	196	25.6
First premolar	112	14.6
Second molar	67	8.7
Second premolar	161	21
Canine	70	9.1
Lateral incisor	85	11.1
Central incisor	76	9.9
Previous Restoration		
Amalgam	103	7.2
Composite	177	12.3
Crown	65	4.5
Bridge	36	2.5
No restoration (cavitated)	1059	73.5
Reason for Endodontic Treatment		
Caries	1174	81.5
Failure of Root Canal Treatment	117	8.1
Periodontal Disease	49	3.4
Prosthetic	81	5.6
Trauma	19	1.4
Apical periodontitis		
AP+	413	28.7
AP-	1027	71.3
Treatment Method		
Root Canal Treatment	1317	91.5
Retreatment	123	8.5

AP: Apical periodontitis

**Table 2:** Descriptive statistics: mean and median values of variables

	Mean ± sd	Median (min-mak)
Age	33.2 ± 13.26	30 (15–79)
Number of Endodontically Treated Teeth	2.08 ± 1.45	2 (1–9)
Number of Missing Teeth	5.14 ± 4.54	4 (1–26)

sd: Standard Deviation, min: Minimum, max: Maximum

### Variables by gender

**Fig. 2:**
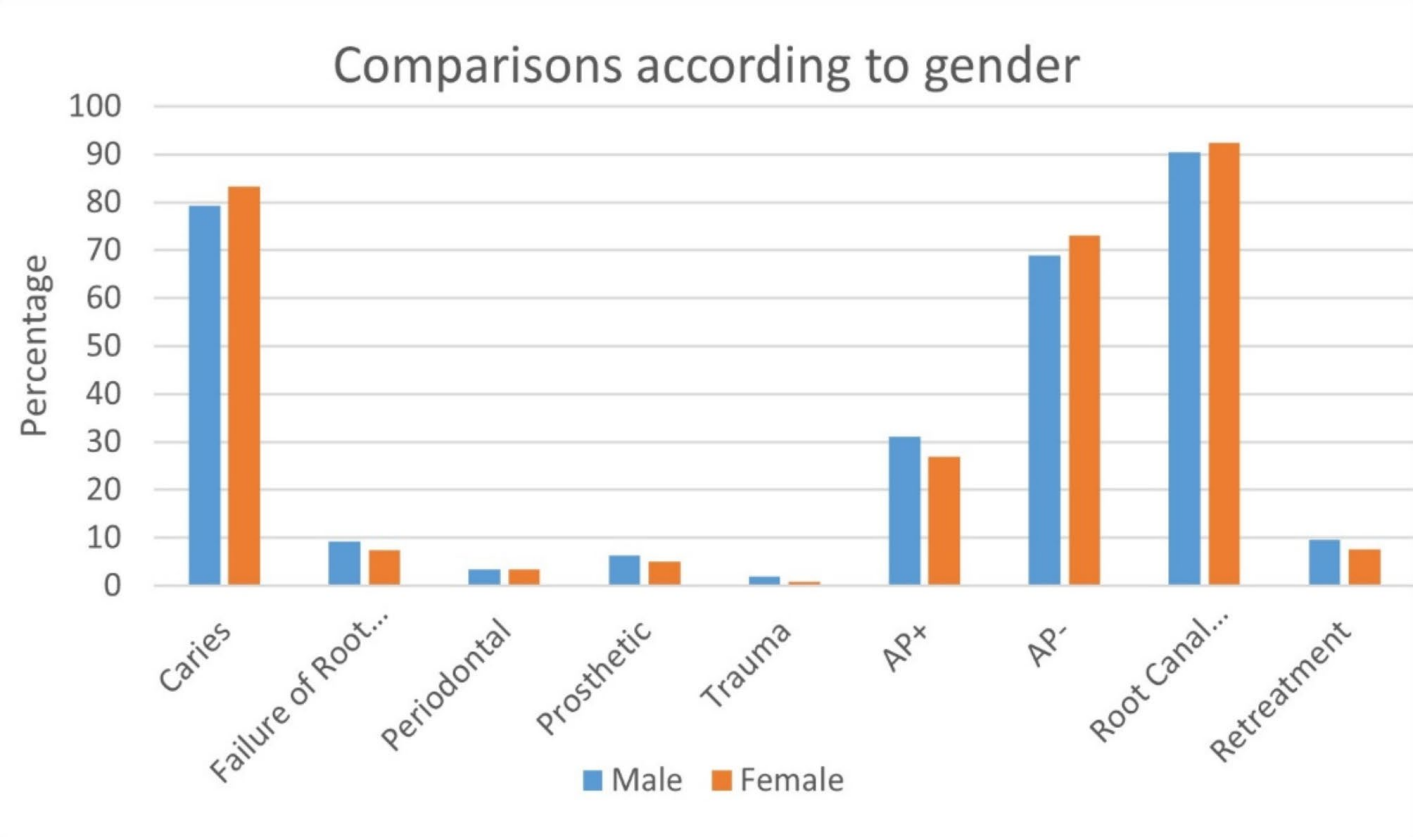
Bar chart of comparisons by gender

A statistically significant difference in the median number of previously endodontically treated teeth was observed between genders (*p* = 0.012). Males had a higher mean rank (357.31) compared to females (321.23). Other variables showed no statistically significant differences by gender (Fig. 2; *p* > 0.05).

### Variables by jaws and tooth position

**Fig. 3:**
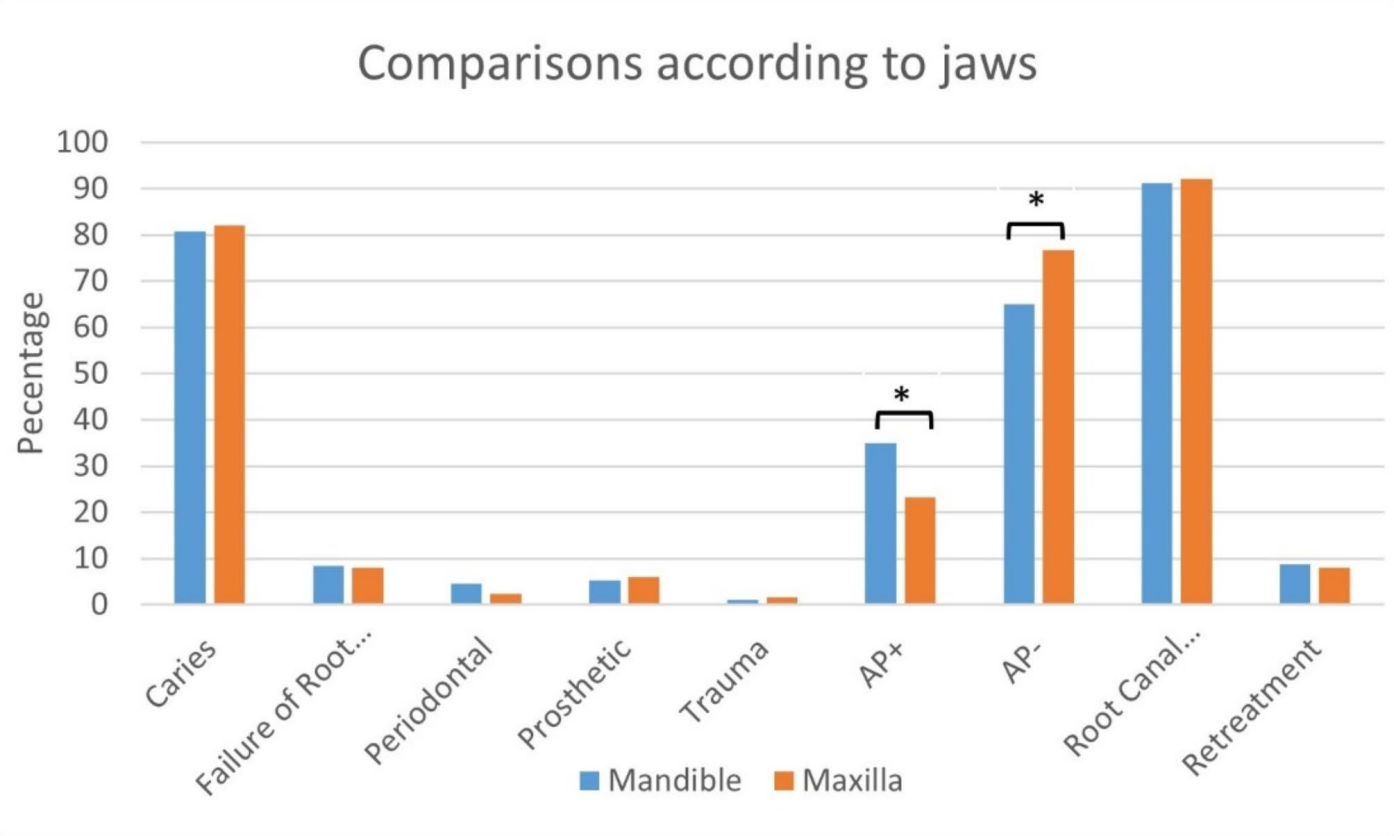
Bar chart of comparisons by jaw. Statistically significant results are indicated by ‘*’. (AP: Apical periodontitis)

The periapical condition of teeth differed significantly between the maxilla and mandible (*p* < 0.001), with a higher lesion rate in mandibular teeth (34.8%) compared to maxillary teeth (23.3%). No significant differences were observed for other variables based on jaw or position (right vs. left) (Fig. 3: *p* > 0.05).

### Mandibular tooth group analysis

Endodontic treatment reasons varied significantly by mandibular tooth group (*p* < 0.001). Caries was more common in molars and premolars, while periodontal disease causes were more frequent in central, lateral, and canine teeth. Prosthodontic reasons were less common in first molars compared to other teeth.

Periapical conditions also differed significantly by mandibular tooth group (*p* < 0.001), with higher lesion rates observed in first molars, first premolars, second molars, and second premolars. Treatment methods and the number of endodontically treated and missing teeth also varied significantly by tooth group (Table 3; *p* = 0.028; *p* = 0.005; *p* < 0.001).

**Table 3:** Comparisons according to tooth group in the mandible

	Tooth Group							Test Statistic	*p*
	First molar	First premolar	Second molar	Second premolar	Canine	Lateral incisor	Central incisor		
Reason for Endodontic Treatment									
Caries	258 (91.8)^a^	47 (81)^abc^	121 (84.6)^ab^	87 (79.8)^bc^	17 (54.8)^bd^	10 (35.7)^d^	4 (17.4)^d^	297.097	< 0.001*
FORCT	19 (6.8)	5 (8.6)	9 (6.3)	15 (13.8)	3 (9.7)	1 (3.6)	4 (17.4)		
Periodontal Disease	3 (1.1)^a^	2 (3.5)^ab^	1 (0.7)^a^	2 (1.8)^a^	6 (19.4)^bc^	11 (39.3)^c^	10 (43.5)^c^		
Prosthetic	2 (0.7)^a^	4 (6.9)^b^	12 (8.4)^b^	5 (4.6)^ab^	6 (19.4)^b^	4 (14.3)^b^	2 (8.7)^b^		
Trauma	0 (0)	0 (0)	0 (0)	0 (0)	0 (0)	4 (14.3)	3 (13)		
Apical periodontitis									
AP+	105 (37.4)^a^	16 (27.6)^a^	38 (26.6)^a^	33 (30.3)^a^	13 (41.9)^ab^	13 (46.4)^ab^	17 (73.9)^b^	25.223	< 0.001*
AP-	176 (62.6)	42 (72.4)	105 (73.4)	76 (69.7)	18 (58.1)	15 (53.6)	6 (26.1)		
Treatment Methods									
Root Canal Treatment	261 (92.9)	53 (91.4)	134 (93.7)	93 (85.3)	27 (87.1)	27 (96.4)	18 (78.3)	13.044	0.042 *
Retreatment	20 (7.1)	5 (8.6)	9 (6.3)	16 (14.7)	4 (12.9)	1 (3.6)	5 (21.7)		
Number of Endodontically Treated Teeth	1.69 ± 1.09	2.45 ± 1.87	2.11 ± 1.48	2.04 ± 1.39	2,89 ± 2.19	3.14 ± 2.57	2,31 ± 1.7	18.638	0.005 **
	1 (1–6)	2 (1–8)	2 (1–8)	2 (1–6)	2 (1–9)	2 (1–9)	2 (1–6)		
Number of Missing Teeth	2.75 ± 2.08	6.54 ± 5.48	3.69 ± 2.88	4.5 ± 3.38	11.06 ± 6.54	11.36 ± 5.97	10.13 ± 6.77	120.514	< 0.001**
	2 (1–13)^d^	5 (1–21)^ce^	3 (1–19)^bd^	3 (1–14)^bc^	10 (1–26)^a^	10 (1–22)^a^	10 (1–23)^ae^		

*Pearson Chi-Square Test; ** Kruskal Wallis Test; a-e: There is no difference between groups with the same letter. FORCT: Failure of Root Canal Treatment

### Maxillary tooth group analysis

Endodontic treatment reasons showed significant variation by maxillary tooth group (Table 4; *p* < 0.001). Caries was more common in molars and second premolars, while periodontal disease causes were more frequent in central and lateral incisors. Prosthodontic reasons were more prevalent in central, lateral, and canine teeth than in second premolars.

**Table 4:** Comparisons according to tooth group in the maxilla

	Tooth Group								Test Statistic	*p*
	First molar	First premolar		Second molar	Second premolar	Canine	Lateral incisor	Central incisor		
Reason for Endodontic Treatment										
Caries	177 (90.3)^ab^		92 (82.1)^bc^	66 (98.5)^a^	144 (89.4)^ab^	50 (71.5)^cd^	56 (65.9)^cd^	45 (59.2)^d^	144.603	< 0.001*
FORCT	15 (7.7)		14 (12.5)	1 (1.5)	13 (8.1)	5 (7.1)	9 (10.6)	4 (5.3)		
Periodontal Disease	2 (1)^a^		0 (0)	0 (0)	1 (0.6)^a^	1 (1.4)^ab^	6 (7.1)^a, b^	8 (10.5)^b^		
Prosthetic	2 (1)^a^		6 (5.4)^a, b,c^	0 (0)	3 (1.9)^a^	12 (17.1)^b^	10 (11.8)^bc^	13 (17.1)^b^		
Trauma	0 (0)		0 (0)	0 (0)	0 (0)	2 (2.9)	4 (4.6)	6 (7.9)		
Apical periodontitis										
AP+	39 (19.9)^a^		18 (6.1)^a^	10 (14.9)^a^	26 (16.1)^a^	11 (15.7)^a^	39 (45.9)^b^	35 (46.1)^b^	118.505	< 0.001*
AP-	157 (80.1)		94 (83.9)	57 (85.1)	135 (83.9)	59 (84.3)	46 (54.1)	41 (53.9)		
Treatment Methods										
Root Canal Treatment	181 (92.3)		98 (87.5)	66 (98.5)	148 (91.9)	65 (92.9)	75 (88.2)	71 (93.4)	8.627	0.196*
Retreatment	15 (7.7)		14 (12.5)	1 (1.5)	13 (8.1)	5 (7.1)	9 (10.6)	4 (5.3)		
Number of Endodontically Treated Teeth	2.04 ± 1.63		2.26 ± 1.51	1.97 ± 1.18	2.05 ± 1.21	2 ± 1.22	2.11 ± 1.22	2.08 ± 1.44	2.908	0.820 **
	1 (1–8)		2 (1–7)	2 (1–5)	2 (1–5)	2 (1–6)	2 (1–6)	1.5 (1–6)		
Number of Missing Teeth	3.02 ± 2.85		4.98 ± 4.1	3.53 ± 2.85	3.95 ± 3.2	8.87 ± 4.14	6.68 ± 4.5	7.15 ± 5.36	122.405	< 0.001**
	2 (1–19)^d^		4 (1–21)^bc^	3 (1–14)^bd^	3 (1–18)^bd^	9 (1–20)^a^	6 (1–23)^ac^	6 (1–21)^ac^		

*Pearson Chi-Square Test; ** Kruskal Wallis Test; a-d: There is no difference between groups with the same letter. FORCT: Failure of Root Canal Treatment

Periapical conditions varied significantly by tooth group (*p* < 0.001), with higher lesion rates in central and lateral incisors and first molars, first premolars, second molars, and second premolars. Missing teeth also differed significantly by tooth group (*p* < 0.001).

### Relationships between variables

A weak but statistically significant positive correlation was found between age and the number of endodontically treated teeth (*r* = 0.090; *p* = 0.019). A moderate positive correlation was observed between age and the number of missing teeth (*r* = 0.618; *p* < 0.001). However, no significant relationship was found between the number of endodontically treated teeth and missing teeth (*p* > 0.05).

### Periapical conditions

Restored teeth had a significantly higher frequency of AP compared to unrestored teeth (*p* < 0.001). The type of restoration was also significantly associated with AP (*p* < 0.001), with crowns having lower lesion rates than bridges and composite restorations (Table 5). AP rates were significantly associated with the reason for endodontic treatment (*p* < 0.001), being lower in teeth treated for prosthetic reasons. Treatment methods also influenced AP rates (*p* < 0.001). The overall AP rate among treated teeth was 28%, with 24% in primary treatments and 74% in retreatments, indicating a higher lesion rate in retreatment cases.

**Table 5:** Distribution of variables according to periapical status

	Apical periodontitis		Test Statistic	p*
	AP+	AP-		
Presence of restoration				
Yes	159 (41.7)	222 (58.3)	42.283	< 0.001*
No	254 (24)	805 (76)		
Previous Restoration				
Amalgam	39 (37.9)^acd^	64 (62.1)	30.388	< 0.001*
Composite	83 (46.9)^ab^	94 (53.1)		
Crown	16 (24.6)^c^	49 (75.4)		
Bridge	21 (60)^bd^	15 (40)		
Reason for Endodontic Treatment				
Caries	274 (23.3)^a^	900 (76.7)	221.139	< 0.001*
Failure of Root Canal Treatment	85 (72.6)^bc^	32 (27.4)		
Periodontal Disease	41 (83.7)^c^	8 (16.3)		
Prosthetic	5 (6.2)^d^	76 (93.8)		
Trauma	8 (42.1)^ab^	11 (57.9)		
Treatment Methods				
Root Canal Treatment	322 (24.4)^a^	995 (75.6)	132.530	< 0.001*
Retreatment	91 (74)^b^	32 (26)		

*Pearson Chi-Square Test; a-d: There is no difference between groups with the same letter

## Discussion

CBCT, panoramic radiographs, and periapical radiographs are commonly used to evaluate AP [5, 12, 27]. CBCT provides three-dimensional imaging, allowing for more accurate assessment of complex anatomical structures, detection of periapical lesions, and evaluation of root canal morphology. Its advantages in diagnosing fractures, resorptions, and endo-perio lesions make it a valuable tool in clinical decision-making [18, 19, 26, 35]. The application of the PAI to CBCT enhances diagnostic reliability by reducing false-negative diagnoses through high-resolution imaging [39]. However, CBCT exposes patients to higher levels of ionizing radiation, limiting its routine use [28]. Panoramic radiographs, while less sensitive, are widely used in epidemiological studies due to their simplicity and broad applicability. In this study, periapical radiographs were employed as they offer superior diagnostic accuracy for detecting periapical radiolucencies compared to panoramic radiographs [40].

This study identified statistically significant differences in the presence of preoperative AP based on tooth position and tooth group, as well as in treatment methods and reasons for treatment among different tooth groups. Additionally, a significant relationship was found between previous restorations, reasons for treatment, treatment methods, and the presence of AP. Based on these findings, the first and second null hypotheses were rejected.

The prevalence of endodontically treated teeth (4.2%) in this study due to previously reported rates ranging from 1.6 to 21% [16, 17, 27, 30, 31, 41]. Similar to other studies [17, 30, 32], no significant gender differences were observed in AP prevalence. However, the higher number of previously endodontically treated teeth in males could be attributed to women’s greater emphasis on dental health and aesthetics, resulting in earlier intervention for caries and pulp diseases [16, 27, 31, 32, 41].

The overall preoperative AP prevalence among the treated teeth in our study was 28%. Retrospective studies in the literature report AP prevalence rates ranging from 2–10% [16, 21, 35]. This discrepancy is primarily because our study evaluated only teeth that underwent treatment, excluding untreated teeth. The reported prevalence in our study reflects only the proportion within treated teeth, while other studies considered all teeth. Our study aimed to guide treatment planning based on treated teeth’ prevalence and preoperative conditions.

Retreatment cases exhibited a higher prevalence of AP compared to primary treatments, consistent with the findings of Tsuneishi et al. and Meirinhos et al. [16, 21, 35, 41]. This is likely due to the persistence of bacterial infections and inadequate prior treatments [42]. Primary treatments had lower AP rates, possibly reflecting early intervention for pulp diseases before periapical involvement [5, 9]. The more frequent application of primary root canal treatment supports this situation. Also, the less frequent retreatment may indicate high survival rates and success of root canal treatment, supporting promising clinical results of endodontic treatment.

AP prevalence was higher in mandibular teeth (34.8%) than in maxillary teeth (23.3%), similar to findings by Kalender et al. [29]. However, other studies have reported mixed findings: Gulsahi et al. found equal AP prevalence in the maxilla and mandible. Tarım Ertaş et al. and Meirinhos et al. reported higher AP prevalence in maxillary teeth. This discrepancy may be explained by higher masticatory forces and complex root canal anatomy in mandibular teeth, making them more susceptible to microcracks and bacterial invasion [35]. The mandibular first molar was the most frequently treated tooth, due to its early eruption and high susceptibility to caries, as previously reported by Sunay et al., Taşsoker et al., and Yousuf et al. [16, 27, 43, 44].

Dental caries was the most common reason for endodontic treatment in this study, particularly in molars. Several studies, including those by Tareen et al., Ahmed et al., and Agrawal et al., have similarly reported that dental caries is the leading cause of endodontic treatment. The deep grooves and fissures of molars, combined with their posterior location, make them more susceptible to food retention, plaque accumulation, and cleaning difficulties [45]. These factors highlight dental caries as a leading cause of tooth loss and pulp irritation, cementing its role as a primary indication for root canal therapy [43]. In contrast, anterior teeth are more prone to trauma, improper brushing, and malalignment, which increase plaque accumulation and tissue loss [46].

Teeth treated for periodontal diseases or previous endodontic failures exhibited higher AP prevalence, likely due to microbial overlap between periodontal and periapical regions and the immune suppression associated with chronic inflammation [47, 48]. Supporting the results of this study, Salceanu et al. and El Ouarti et al. found that the presence of periodontal disease contributes to a higher prevalence of AP [49, 50]. Additionally, inadequate root canal treatments significantly contributed to AP rates, highlighting the association between treatment failure and retreatment cases [42].

The persistence of caries and periodontal diseases underscores the inadequacy of preventive dental practices in addressing oral health comprehensively. Despite advancements in dental care, these conditions remain prevalent, emphasizing the need for accessible and effective preventive strategies [51, 52]. Public awareness campaigns and routine preventive measures could substantially reduce the burden of endodontic interventions.

Teeth with restorations demonstrated higher AP prevalence compared to unrestored teeth, likely due to thermal and mechanical trauma during preparation, marginal leakage, or secondary caries [48]. Bridge restorations were associated with a higher AP risk than crowns, possibly due to increased mechanical stress and plaque accumulation under the bridge [48, 53]. In contrast, crowns with proper marginal adaptation, occlusal fit, and absence of secondary caries or marginal leakage have been shown to improve the long-term success of endodontically treated teeth by preventing reinfection [54, 55].

Taşsoker et al. and Pietrzycka et al. reported that the number of endodontically treated and missing teeth increases with age [17, 18]. This cumulative effect can be attributed to the rising prevalence of caries, trauma, and periodontal diseases, along with prolonged exposure to functional and iatrogenic factors [18]. Changes in pulp defense and reparative capacity with age may also influence the frequency of endodontic treatment [56]. This study also found that the number of endodontically treated and missing teeth increased with age. However, there was no significant correlation between endodontically treated teeth and missing teeth. Literature suggests that endodontic treatment does not necessarily reduce the fracture resistance of teeth and that, in cases where an adequate ferrule is present, both direct and indirect restorations exhibit similar mechanical performance to non-endodontically treated teeth [57, 58]. Therefore, the third null hypothesis is accepted. This supports the idea that endodontic treatment is crucial in preserving tooth survival and reducing the need for extractions.

A higher prevalence of AP was observed in incisors compared to other tooth groups in both the maxilla and mandible. Although many studies have shown that AP prevalence is higher in molar teeth [16, 31, 35], Monteiro et al. reported a higher AP prevalence in maxillary incisors [59]. This could be attributed to patients’ preference for extraction over treatment in molar or premolar regions when AP is present. In contrast, anterior teeth are often prioritized for treatment due to concerns about aesthetic appearance, leading patients to seek endodontic treatment instead of extraction.

Discrepancies in the results of epidemiological studies may stem from methodological differences, population diversity, participants’ treatment preferences, educational background, and socioeconomic status [35, 43, 60].

One of the key strengths of this study is the use of radiographs taken during treatment planning, enabling accurate diagnosis of AP and confirmation of appropriate endodontic indications. Preoperative AP cases were definitively diagnosed as active, rather than being in a healing phase. Additionally, radiographic evaluations were conducted using both panoramic and periapical radiographs for each treated tooth. This methodological approach minimizes assumptions regarding AP, offering a significant advantage over many studies in the literature. Consequently, the data obtained are highly reliable and reflective of real-world clinical scenarios.

A notable limitation of this study is its single-center design, conducted in Van, Turkey. Additionally, the study population was drawn exclusively from patients attending a public hospital, introducing the potential for selection bias. Furthermore, in clinical situations where CBCT imaging is available, the reproducibility of results should be evaluated, considering CBCT’s superiority in assessing periapical pathology and treatment variables. Future research should aim to overcome these limitations by incorporating multicenter studies with participants from diverse regions across Turkey. This approach would provide a more comprehensive understanding of endodontic treatment needs and outcomes.

## Conclusion

This study is among the few to examine epidemiological factors associated with preoperative AP in a Turkish subpopulation, offering valuable regional data that enhance the global understanding of endodontic treatment outcomes. Unlike previous research focused primarily on overall endodontic success rates, this study identifies specific risk factors for preoperative AP, underscoring the importance of early intervention in treatment planning. The findings reveal that preoperative restorations, periodontal disease, failed root canal treatments, and retreatment cases increase the incidence of AP, whereas crown restorations appear to have a protective effect. This highlights the need for comprehensive preoperative assessment and tailored treatment strategies to improve patient outcomes. Integrating preventive measures, such as timely restorative interventions and regular follow-ups, can reduce the need for retreatment and enhance long-term success. Additionally, while the number of endodontically treated and missing teeth increases with age, no statistically significant relationship was found, suggesting that successful endodontic treatment contributes to dentition preservation.

## Data Availability

If the data is requested with justification, it will be sent by the authors.

## Abbreviations

AP: Apical periodontitis
CBCT: Cone-beam computed tomography
Cm: Centimeter
cm^2^: Square centimeter
kV: Kilovolt
kVP: Kilovoltage
mA: Miliamper
PAI: Periapical index
